# Evaluation of direct inoculation of the BD PHOENIX system from positive BACTEC blood cultures for both Gram-positive cocci and Gram-negative rods

**DOI:** 10.1186/1471-2180-11-156

**Published:** 2011-06-30

**Authors:** Judith Beuving, Christina FM van der Donk, Catharina FM Linssen, Petra FG Wolffs, Annelies Verbon

**Affiliations:** 1 Department of Medical Microbiology, Care And Public Health Research Institute (CAPHRI), Maastricht University Medical Center, PO Box 5800, 6202AZ, Maastricht, the Netherlands; 2 Department of Internal Medicine, Erasmus University Medical Center, PO Box 2040, 3000CA, Rotterdam, the Netherlands

## Abstract

**Background:**

Rapid identification (ID) and antibiotic susceptibility testing (AST) of the causative micro-organism of bloodstream infections result in earlier targeting of antibiotic therapy.

In order to obtain results of ID and AST up to 24 hours earlier, we evaluated the accuracy of direct inoculation of the Phoenix system from positive blood cultures (BACTEC) by using Serum Separator Tubes to harvest bacteria from positive blood cultures. Results were compared to those of standard Phoenix procedure. Discrepancies between the two methods were resolved by using the API system, E-test or microbroth dilution.

**Results:**

ID with the direct method was correct for 95.2% of all tested *Enterobacteriaceae *(n = 42) and 71.4% of *Pseudomonas aeruginosa *strains (n = 7).

AST with the direct method showed a categorical agreement for Gram-negative rods (GNR) of 99.0%, with 0.7% minor errors, 0.3% very major errors and no major errors. All antibiotics showed an agreement of >95%.

The direct method for AST of *Staphylococcus *(n = 81) and *Enterococcus *(n = 3) species showed a categorical agreement of 95.4%, with a minor error rate of 1.1%, a major error rate of 3.1% and a very major error rate of 0.4%. All antibiotics showed an agreement of >90%, except for trimethoprim-sulfamethoxazole and erythromycin.

**Conclusions:**

Inoculation of Phoenix panels directly from positive blood cultures can be used to report reliable results of AST of GNR a day earlier, as well as ID-results of *Enterobacteriaceae*. For *Staphylococcus *and *Enterococcus *species, results of AST can also be reported a day earlier for all antibiotics, except for erythromycin and trimethoprim-sulfamethoxazole.

## Background

Bloodstream infections are a common condition, affecting approximately 2% of all hospitalised patients and up to 70% of all patients in the Intensive Care Unit, and the incidence is rising [1-4]. Mortality is high, ranging from 14 to 57% [5]. In this group of patients, rapid identification (ID) and antibiotic susceptibility testing (AST) of the causative microorganism are essential since they result in earlier targeting of antibiotic therapy [6-9]. Early administration of adequate antibiotic therapy has been shown to reduce mortality [10-12].

The introduction of automated blood culture systems and automated systems for ID and AST have reduced the time to diagnosis in bloodstream infections. However, for these systems, blood cultures have to be subcultured on agar before ID and AST can be performed, which can take up to 24 hours.

An alternative for subculture on agar is harvesting the bacteria needed for inoculation of these systems directly from positive blood cultures by using Serum Separator Tubes, thereby reducing the time needed to obtain results of ID and AST by a day. Although this method has been successfully tested for many automated systems [13-17], direct inoculation was reported only twice for the BD Phoenix Automated Microbiology System (BD), once for Gram-negative rods (GNR) [18] and once for Gram-positive cocci (GPC) [19]. Both studies compared their results of the direct method with results of the Vitek system.

No studies are available comparing results of direct inoculation with the routinely used method of inoculating the Phoenix system, which is the standard procedure for ID and AST in many microbial diagnostic laboratories. Here, we evaluated the accuracy of direct inoculation of the Phoenix system with positive blood culture isolates, compared to the routinely used procedure.

## Methods

### Sample collection

Between January and April 2009, blood cultures grown in the previous 24 hours in the Bactec automated blood culture device (Bactec™ 9240, BD Diagnostic Systems, Sparks, MD, USA) and containing *Staphylococcus *species, *Enterococcus *species or obligate aerobic and facultative anaerobic GNR were evaluated. Polymicrobial cultures as well as cultures containing anaerobes or fungi were excluded from the analysis. *Streptococcus *spp. are not routinely processed in the Phoenix system in our lab and were therefore also excluded from the analysis. One positive blood culture per patient per episode of bloodstream infection was included in the study. The study was performed in the Department of Medical Microbiology of the Maastricht University Medical Center (MUMC), a 750-bed referral hospital.

All samples were used according to the code for proper use of human tissue as formulated by the Dutch Federation of Medical Scientific Societies.

### Blood cultures

Blood drawn from patients admitted in the MUMC and suspected for bloodstream infection was incubated in blood culture bottles (Plus+Aerobic (product no. 442192; BD) and Plus+Anaerobic (product no. 442193; BD)) and monitored for microbial growth in the Bactec™ 9240 instrument (BD). When growth was detected by the instrument, Gram-staining was performed.

### Direct inoculation

For the direct method, 5 ml of grown blood culture was aspirated from the blood culture bottle and the aspirate was injected in a Serum Separator Tube (SST) (BD Diagnostic Systems, Sparks, MD, USA). This tube was centrifuged at 2000 × *g *for 10 minutes, after which the supernatant was discarded. Bacteria were harvested from the gel layer using a sterile cotton swab and suspended in a Phoenix system ID broth tube (product no. 246000; BD) until a 0.5 McFarland standard suspension was obtained. To obtain optimal results, for Gram-negative isolates, 25 μl of this suspension were transferred into a tube of Phoenix system AST broth (product no. 246002; BD) in which one drop of AST indicator (product no. 246004; BD) was previously added, according to manufacturers' guidelines. Since the inoculum of GPC in ID broth was shown to be almost 10 times lower than is standard in a 0.5 McFarland suspension, 250 μl of inoculated ID broth was added to AST broth for GPC, instead of the 25 μl in the manufacturers' guidelines. For GNR, the Phoenix system panel NMIC/ID-75 (product no. 448087; BD) was used. For GPC, the PMIC-58 panel (product no. 448052; BD) was used.

To calculate the original number of CFU/ml in the ID broth and to serve as purity control, dilutions of ID broth were also subcultured, using the Eddy Jet spiral plater (IUL, S.A., Barcelona, Spain).

### Routinely used inoculation

For the routinely used method, a small volume of positive blood culture was inoculated on Columbia sheep blood agar plates and incubated at 35°C with 5% CO_2_. A standard inoculum in ID broth was prepared from the bacteria grown on the agar medium and inoculated into Phoenix panels, following the manufacturer's recommendations.

### Identification of GPC

Since the Phoenix system is not used for ID of GPC in routine diagnostics, ID by direct inoculation was not tested in this group. To discern *Staphylococcus *species from other GPC, a catalase test was performed. For the identification of *Staphylococcus *species, catalase-positive strains were tested for coagulase and DNAse production. If both tests were negative, the strain was identified as a coagulase-negative *Staphylococcus *species (CoNS).

To discern *Enterococcus *species from other catalase-negative GPC, bile esculin and tellur diagnostic tablets (Rosco Diagnostica, Taastrup, Denmark) were used, according to manufacturer's guidelines. If both tests were positive, the strain was identified as *Enterococcus faecalis*, whereas in case of a positive bile esculin test but a negative tellur test, an API 20 Strep test (Biomérieux SA, Marcy l'Etoile, France) was performed to further identify the strain.

Results of identification were adjusted in the Phoenix results retrospectively for both the standard and direct method, after which the software automatically adjusted MIC cutoff values to those of the identified species.

### Discrepancy analysis

To resolve differences in ID of GNR, the API system was used (API 20E for *Enterobacteriaceae *and API 20NE for non-fermenters (Biomérieux)). In case of discrepancies in AST between results of the direct method and the routinely used method for ceftazidime, ceftriaxone, cefuroxime, ciprofloxacin, clindamycin, levofloxacin, moxifloxacin, linezolid, penicillin, piperacillin, piperacillin-tazobactam, and tobramycin an E-test (Biomérieux) was performed according to manufacturer's guidelines, and used as gold standard [20,21]. Discrepancies for amoxicillin, amoxicillin-clavulanate, erythromycin, gentamicin, oxacillin, rifampin, tetracycline and trimethoprim-sulfamethoxazole were resolved using microbroth dilution, as described in the CLSI-guidelines [22].

### Data analysis

Results of AST with the direct method (susceptible, intermediate or resistant) were analysed for categorical agreement with the results of the standard method. Also, minor errors (any false result involving an intermediate result), major errors (false-resistant results) and very major errors (false-susceptible results) were calculated.

### Statistical analysis

Bacterial load in ID broth for GPC and GNR was compared using an independent samples t-test.

## Results

### Inoculum of bacteria in ID broth after use of serum separator tubes (SSTs)

In total, 134 blood cultures were included, from 116 patients. The inoculum of GPC in ID broth was on average 3.6 × 10^7 ^CFU/ml, whereas that of GNR was 1.8 × 10^8 ^CFU/ml, which was a significant difference (95% CI between -1.7 × 10^8 ^and -1.2 × 10^8^; P < 0.001).

### ID of GNR with the direct Phoenix method

ID with direct inoculation was correct for 95.2% of all tested *Enterobacteriaceae*. One *Escherichia coli *strain was incorrectly identified as *Salmonella choleraesuis *with the direct method. One *Serratia marcescens *strain could not be identified with the direct method. Identification for *Pseudomonas *spp. was correct in 71.4%. Both errors in this group involved strains of *Pseudomonas aeruginosa *that were incorrectly identified as *Pseudomonas fluorescens *(Table 1). No errors in ID were observed for the routine method.

**Table 1:** Results of identification of GNR with the direct method

	Total no. of strains	No. of unidentified strains	No. of misidentified strains	ID of misidentified strains
***Enterobacteriaceae***				
*E. coli*	26		1	*Salmonella choleraesuis*
*K. pneumoniae spp. pneumoniae*	8			
*S. marcescens *	4	1		
*K. oxytoca *	1			
*P. mirabilis *	1			
*E. cloacae *	1			
*M. morganii *	1			
**Non-fermenters**				
*P. aeruginosa *	7		2	*Pseudomonas fluorescens*

### Antibiotic susceptibility testing (AST) of GNR

Results of AST were available for 49 strains, one *P. aeruginosa *strain failed to grow sufficiently in the Phoenix system so no results were available for the direct method. Categorical agreement of the direct method with results of the standard method for GNR was 97.6%. After discrepancy analysis of the results of AST, this percentage rose to 99.0%, with 5 minor errors (0.7%), no major errors, and 2 very major errors (0.3%) (Table 2). Both very major errors occurred with trimethoprim-sulfamethoxazole in *Pseudomonas aeruginosa *strains. Categorical agreement of the standard method after discrepancy analysis was 98.4% (table 2). One very major error occurred with trimethoprim-sulfamethoxazole. No antibiotic showed a categorical agreement of <95% (Table 3).

**Table 2:** Agreements and errors for AST of GPC and GNR for the direct and routinely used Phoenix method

	Direct vs routinely used method	Direct method after discrepancy analysis	Routine method after discrepancy analysis
**GPC (n = 84)**			
Categorical agreement	93.1%	95.4%	97.3%
Minor errors	1.7%	1.1%	0.7%
Major errors	4.2%	3.1%	0.8%
Very major errors	0.9%	0.4%	1.6%

**GNR (n = 49)**			
Categorical agreement	97.6%	99.0%	98.4%
Minor errors	1.9%	0.7%	1.4%
Major errors	0.1%	0.0%	0.1%
Very major errors	0.4%	0.3%	0.1%

**Table 3:** Agreement and errors of the direct method for AST for GNR, after discrepancy analysis

Antimicrobial agent	No. of tested strains	% categorical agreement	No. of minor errors (%)	No. of major errors (%)	No. of very major errors (%)
Amikacin	49	100	0	0	0
Amoxicillin/clavulanate	49	98.0	1 (2.0)	0	0
Ampicillin	49	98.0	1 (2.0)	0	0
Ceftazidime	49	100	0	0	0
Ceftriaxone	49	98.0	1 (2.0)	0	0
Cefuroxime	49	98.0	1 (2.0)	0	0
Ciprofloxacin	49	100	0	0	0
Colistin	49	100	0	0	0
Gentamicin	49	100	0	0	0
Levofloxacin	49	100	0	0	0
Meropenem	49	100	0	0	0
Piperacillin	49	98.0	1 (2.0)	0	0
Piperacillin/tazobactam	49	100	0	0	0
Tobramycin	49	100	0	0	0
Trimethoprim/sulfamethoxazole	49	96.0	0	0	2 (4.0)

Total	735	99.0	5 (0.7)	0 (0)	2 (0.3)

### AST of GPC

AST using the direct method was performed for 84 GPC (22 *Staphylococcus aureus*, 59 CoNS, 2 *Enterococcus faecalis *and 1 *Enterococcus faecium*). Categorical agreement for the tested GPC was 93.1% compared with results of the standard method. After discrepancy analysis this was 95.4%, with a minor error rate of 1.1%, a major error rate of 3.1% and a very major error rate of 0.4% (Table 2). Except for erythromycin and trimethoprim-sulfamethoxazole, all antibiotics showed a categorical agreement of the direct method of >90% (table 4). Again, all very major errors (n = 4) occurred with trimethoprim-sulfamethoxazole, all in CoNS strains. The major errors were divided as follows: 10 for *S. aureus*, 23 for CoNS and 1 for *Enterococcus *spp..

**Table 4:** Agreement and errors of the direct method of AST for GPC after discrepancy analysis

Antimicrobial agent	No. of tested strains	% categorical agreement	No. of minor errors (%)	No. of major errors (%)	No. of very major errors (%)
Amoxicillin/clavulanate	84	91.7	0.0	7 (8.3)	0
Ampicillin	84	94	0	5 (6.0)	0
Clindamycin	84	96.4	2 (2.4)	1 (1.2)	0
Erythromycin	84	86.9	8 (9.5)	2 (3.6)	0
Gentamicin	84	100	0	0	0
Linezolid	84	91.6	1 (1.2)	6 (7.2)	0
Moxifloxacin	84	100	0	0	0
Oxacillin	84	96.4	0	3 (3.6)	0
Penicillin	84	98.8	0	1 (1.2)	0
Rifampin	82	98.8	0	1 (1.2)	0
Tetracycline	84	97.6	1 (1.2)	1 (1.2)	0
Trimethoprim/Sulfamethoxazole	84	89.2	0	5 (6.0)	4 (4.8)
Vancomycin	84	98.8	0	1 (1.2)	0

Total	1090	95.4	12 (1.1)	34 (3.1)	4 (0.4)

Categorical agreement for the standard method after discrepancy analysis was 97.3% (see table 2). One very major error occurred for amoxicillin-clavulanate, 1 for ampicillin, 1 for erythromycin, 4 for gentamicin, 1 for moxifloxacin, 2 for oxacillin, 1 for tetracycline and 3 for trimethoprim-sulfamethoxazole (Table 4).

## Discussion

This study shows SSTs can be used to inoculate Phoenix ID broth to a 0.5 McFarland standard, as was also shown by Funke *et al. *for GNR [18]. However, a 0.5 McFarland standard for GPC obtained by using SSTs was shown to consistently contain a lower inoculum than 1.5 × 10^8 ^CFU/ml. This may be due to the presence of blood culture components other than bacteria, since they could also contribute to the turbidity in the ID broth.

This study shows very good results for ID of *Enterobacteriaceae*. Only two errors occurred with ID in this group. One strain was not identified and one strain of *E. coli *was misidentified as *S. choleraesuis*. Results of ID for *Pseudomonas *species were less reliable. Both errors in this group were *P. aeruginosa *strains that were identified as *P. fluorescens*, a rare cause of bloodstream infections. These misidentifications did not lead to errors in interpretation of AST, but rare or unlikely results of ID should be dealt with carefully and be confirmed using additional tests. Other studies also showed that ID of non-fermenting GNR was less reliable than that of *Enterobacteriaceae *[18,23]. This may be due to the lower growth rate of non-fermenters, which could result in weaker fluorescent biochemical reactions in the Phoenix ID panel. Errors in ID with the direct method could also be caused by traces of blood culture components in the ID broth. This however seems less likely, since with *Enterobacteriaceae*, errors in ID were rare.

Since the Phoenix system was not used for ID of GPC, ID by direct inoculation was not tested in this group. But since ID is required for interpretation of AST, in clinical practice, rapid AST will have to be combined with a rapid method of ID, such as PCR-based methods on whole blood, like LightCycler^® ^SeptiFast Test MGRADE (Roche), VYOO Sepsis Test (SIRS-Lab), SepsiTest™ (Molzym), or MALDITOF-MS on positive blood cultures [24].

Some studies on direct methods for AST showed poor results for GPC [15,16] or focus on GNR only due to unfavorable results for GPC [17]. However, in this study, direct AST for *Staphylococcus *species and *Enterococcus *species showed good agreement with conventional methods, comparable to results of the standard method, but with fewer very major errors. Lupetti *et al. *[19], who tested the direct Phoenix method for GPC and compared their results with those of the Vitek 2, found an even higher agreement. They incubated a portion of the positive blood culture with saponin in order to harvest more bacteria from a positive blood culture through the release of intracellular bacteria.

Other studies that presented results of direct methods for AST of GPC showed variable results [13-16,25,26], which makes comparison difficult. But our results were comparable to those of the routine Phoenix method. Moreover, categorical agreement for most tested antibiotics in this study, including oxacillin and vancomycin, were well over 90% and the percentage of major and very major errors is low, meeting the standards proposed by Jorgensen *et al. *[27]. Only erythromycin and trimethoprim-sulfamethoxazole showed lower agreements. The majority of errors for erythromycin were minor errors, but also some major errors occurred. Trimethoprim-sulfamethoxazole was the only antibiotic for both GPC and GNR showing very major errors. Lupetti *et al. *showed a lower agreement of 94% for erythromycin as well, but observed no very major errors for trimethoprim-sulfamethoxazole. Some other studies on direct methods for AST showed some very major errors for trimethoprim-sulfamethoxazole [15,16,18], but only Kerremans *et al. *[13] found a very high percentages of very major errors for this antibiotic in GPC, but not in GNR. Therefore, we conclude that the direct Phoenix method using SSTs can be used to reliably report results of AST for GPC, except for trimethoprim-sulfamethoxazole and erythromycin.

The direct method of AST for GNR showed very good agreement with conventional methods for both *Enterobacteriaceae *and *Pseudomonas *species, comparable to the routinely used method, with essential agreements and categorical agreements of over 95% for all antibiotics tested (see table 3). Both very major errors occurred with trimethoprim-sulfamethoxazole in *Pseudomonas aeruginosa *strains that were correctly identified. For these strains, it would never be considered an adequate treatment, due to intrinsic resistance. These errors thus would not have clinical consequences. Funke *et al. *[18] also described a categorical agreement of 99.0%, which is comparable with or higher than results from studies on other direct methods of AST [7,13-16,26]. Therefore, we conclude that also for GNR, results of the direct Phoenix method for AST can be used to guide antibiotic therapy in bloodstream infections.

The strains tested in this study are a representative sample of the strains most frequently encountered in clinical practice. A limitation of the study is the low number of tested *Enterococcus *and *Pseudomonas *strains (3 and 7, respectively), however, both groups show very good agreement, with only few errors.

Inoculating ID and AST broth by using SSTs can be performed as soon as blood culture bottles are taken out of the BACTEC system and takes approximately 30 minutes, whereas a subculture takes up to 24 hours. Therefore, by using the direct method, results of ID and AST can be available up to 23.5 hours earlier than with the routinely used method.

## Conclusions

From these results we conclude that AST by inoculating Phoenix panels with bacteria harvested directly from positive blood culture bottles is as reliable as using bacteria from a subculture on agar, with the exception of results for erythromycin and trimethoprim-sulfamethoxazole in *Staphylococcus *and *Enterococcus *spp., which should not be reported due to their low agreement. Results of ID of *Enterobacteriaceae *were shown to be very reliable. ID of *Staphylococcus *and *Enterococcus *spp. was not performed with the direct method. Caution is warranted about interpretation of results of *Enterococcus *and *Pseudomonas *spp., of which only a limited number of strains was tested.

Thus, by only a small change in daily laboratory routine, results of ID and AST of positive blood culture isolates can be obtained up to a day earlier than with the standard method, thereby leading to earlier targeting of antibiotic therapy in patients with bloodstream infections.

## Authors' contributions

JB: conceived of the study, performed the gold standard tests and statistical analysis, and drafted the manuscript. CFMD: carried out the direct Phoenix method, performed the analysis and helped to draft the manuscript. CFML: participated in the design of the study and helped to draft the manuscript. PFGW: participated in the design of the study and helped to draft the manuscript. AV: conceived of the study, coordinated it, and helped to draft the manuscript.

All authors read and approved the final manuscript.
